# The Ethics of Pain

**Published:** 1886-03

**Authors:** 


### THE ETHICS OF PAIN.

With a vast capacity for pain, man seems to contain no machinery designed to produce it. It has always the air of a disturbance or an intrusion. Yet, although not omnipresent, it is every imminent, and by it the energies which are most proper to man are suspended or rendered difficult. It often sweeps away the resolves of the will and dictates its own terms to our moral nature. We can account for the whole of bodily pleasure on physical grounds, but for only a portion of bodily pain. To our moral sphere of being, therefore, as I hope to show more fully further on, we must look for a full account of it. If the maximum of voluptuous enjoyment had been the highest object of our nature, still, even so, room would be found for pain in the animal economy, since in order for existence to be enjoyed it must first be preserved. By pain felt or apprehended we are protected from fatal hurts or warned of their existence, and so with regard to the approaches or the fact of diseased action in the body. And this to a large extent animals share with us. Pleasure seems to have no such preservative element in it, and our capacity for it might be conceived so enlarged as to be a constant blind to precaution and lure to destruction. Pain, on the other hand, is largely charged (as shown above) with a preservative power. An enlarged capacity of pleasure would be certainly followed by an increased pursuit of it. On the contrary, enlarged capacity for pain need not imply the increase of actual pain in proportion. Experience, reflection, and precaution, being elements of our nature, might even make a much less amount of pain actually endured consistent with that enlarged capacity. And to whatever extent these elements of our nature could succeed in averting pain, some such rule of inverse proportion would probably result. But the tendency then would be to absorb an undue part of our energies in constant manœuvring to avoid pain. This is neutralized by there being kinds and degrees of pain which cannot be so avoided, although few which are not open to some remedial alleviation.—*The British Quarterly Review.*

**Professor A. B. Palmer**, discussing the action of alcohol upon the lungs, declares there are no statistics—no recorded observations and comparison of numbers of cases—which afford the slightest indication that the use of alcohol in any form or quantity prevents consumption. It grows more and more difficult to find really unassailable excuses for liquor drinking. The consumption cure theory has had a long service, and now that it has been annihilated it will be hard for the topers to devise one as useful.

